# Enhanced NAMPT-Mediated NAD Salvage Pathway Contributes to Psoriasis Pathogenesis by Amplifying Epithelial Auto-Inflammatory Circuits

**DOI:** 10.3390/ijms22136860

**Published:** 2021-06-25

**Authors:** Laura Mercurio, Martina Morelli, Claudia Scarponi, Giovanni Luca Scaglione, Sabatino Pallotta, Daniele Avitabile, Cristina Albanesi, Stefania Madonna

**Affiliations:** 1 Laboratory of Experimental Immunology and Dermatology Division, IDI-IRCCS, I-00167 Rome, Italy; l.mercurio@idi.it (L.M.); martina.morelli@idi.it (M.M.); c.scarponi@idi.it (C.S.); g.scaglione@idi.it (G.L.S.); s.pallotta@idi.it (S.P.); c.albanesi@idi.it (C.A.); 2 IDI-Farmaceutici S.r.l. Pomezia, I-00171 Rome, Italy; davitabile@idifarmaceutici.it

**Keywords:** NAMPT, visfatin, NAD salvage pathway, resident skin cells, psoriasis, inflammation, keratinocytes

## Abstract

Dysregulated cross-talk between immune cells and epithelial compartments is responsible for the onset and amplification of pathogenic auto-inflammatory circuits occurring in psoriasis. NAMPT-mediated NAD salvage pathway has been recently described as an immunometabolic route having inflammatory function in several disorders, including arthritis and inflammatory bowel diseases. To date, the role of NAD salvage pathway has not been explored in the skin of patients affected by psoriasis. Here, we show that NAD content is enhanced in lesional skin of psoriatic patients and is associated to high NAMPT transcriptional levels. The latter are drastically reduced in psoriatic skin following treatment with the anti-IL-17A biologics secukinumab. We provide evidence that NAMPT-mediated NAD^+^ metabolism fuels the immune responses executed by resident skin cells in psoriatic skin. In particular, intracellular NAMPT, strongly induced by Th1/Th17-cytokines, acts on keratinocytes by inducing hyper-proliferation and impairing their terminal differentiation. Furthermore, NAMPT-mediated NAD^+^ boosting synergizes with psoriasis-related cytokines in the upregulation of inflammatory chemokines important for neutrophil and Th1/Th17 cell recruitment. In addition, extracellular NAMPT, abundantly released by keratinocytes and dermal fibroblasts, acts in a paracrine manner on endothelial cells by inducing their proliferation and migration, as well as the expression of ICAM-1 membrane molecule and chemokines important for leukocyte recruitment into inflamed skin. In conclusion, our results showed that NAMPT-mediated NAD salvage pathway contributes to psoriasis pathogenic processes by amplifying epithelial auto-inflammatory responses in psoriasis.

## 1. Introduction

The cofactor nicotinamide adenine dinucleotide (NAD) is an important metabolic regulator of cellular redox reactions, and acts as a co-factor or co-substrate for key enzymes essential for normal cellular functions in different tissues [1,2]. Intracellular NAD^+^ can be produced de novo from tryptophan via kynurenine pathway or from nicotinic acid (NA) via the Preiss-Handler pathway [3]. However, the ample bulk of NAD in cells is predominately generated via NAD salvage pathway starting from the precursor molecule nicotinamide (NAM) [4]. 

NAM originates from the diet or can be produced by the activity of a variety of NAD hydrolase enzymes, including sirtuins, PARPs, and CD38, which are strictly coupled with the salvage pathway and influence inflammation, cell growth, and bioenergetics [5]. These enzymes degrade NAD and generate NAM as a by-product [6,7]. 

It is known that NAD levels decline with age in different tissues, including skin, due to reduced activities of both salvage and de novo NAD synthesis pathways. Growing evidence also show that certain NAD hydrolases, including CD38, increases in specific tissues during aging and reduces further the intracellular NAD content [8,9,10]. Viceversa, increased NAD levels are associated with inflammatory conditions, including cancer [11]. 

Evidence from animal studies indicates that interventions increasing NAD levels produce numerous benefits on the overall cardio-metabolic health and immune function [2]. Regarding skin conditions, randomized controlled clinical trials have shown that NAM reduces the development of new non-melanoma skin cancers in high-risk humans [12]. However, the mechanism(s) by which NAM could prevent non-melanoma skin cancers remain to be established. 

NAD synthesis in mammalian cells largely depends on the salvage pathway [13], during which NAM is converted to NAD^+^ by two successive reactions involving the enzymes NAMPT and NMNAT, respectively [14]. The key step in this process is the conversion of NAM to NMN through the rate-limiting activity of NAMPT [14].

Although NAMPT functions as an intracellular NAD synthesis enzyme (iNAMPT) in cells, an extracellular form of NAMPT (eNAMPT, also known as visfatin) exists, which is secreted released by several cell types, including pancreatic β-cells, myocytes, and hepatocytes [15,16,17]. It is reported that eNAMPT is also abundantly produced and secreted by adipocytes in visceral fat, with its circulating levels increasing concomitantly to obesity or type 2 diabetes [18,19]. Other than binding to insulin receptors, eNAMPT is considered as an inflammatory adipokine, being able to enhance the production of IL-6, TNF-α, and IL-1 in human monocytes, macrophages, and dendritic cells [18]. It is reported that eNAMPT also promotes angiogenesis by inducing the secretion of CCL2, VEGF, and MMP-2 by human endothelial cells [20,21,22,23,24,25], whereas it enhances CXCL8, CXCL10, and CCL20 release by human keratinocytes [26]. To date, limited data regarding the role of NAD metabolism in skin of patients affected by psoriasis exist. 

Psoriasis is a chronic inflammatory skin disease characterized by erythematous plaques and associated with numerous co-morbidities, including arthritis, cardiovascular disease, overweight/obesity, and inflammatory bowel disease [27,28]. Its pathogenesis comprises a predominant infiltrate of innate immune cells and inflammatory Th1/Th17 lymphocytes releasing IL-17, IFN-γ, and TNF-α cytokines, hyperproliferation and impaired differentiation of epidermal keratinocytes, as well as increased dermal angiogenesis [29,30]. Cross-talk between immune cells and epithelial compartments, which results in the release of cytokines, chemokines, and growth factors, is responsible for the onset and amplification of pathogenic auto-inflammatory circuits, leading to the exacerbation of the disease [30]. 

A study devoted to determining in vivo NAD level in psoriatic skin *showed* reduced NADH amounts in psoriatic lesions [31]. Viceversa, a previous in vitro study demonstrated that total NAD content is significantly increased by about 30% in both subcorneal and basal epidermal layers of psoriatic skin lesions, compared to uninvolved or healthy skin biopsies [32]**.** Although previous studies showed that the combination of NAM with calcipotriene may provide additional benefit in the topical treatment for patients with psoriasis [33,34], the effective efficacy of NAM in psoriasis management remains to be established. 

In this study, we started from the observation on increased amounts of NAD pool in lesional (LS) skin of patients with plaque psoriasis, compared to uninvolved skin. These data prompted us to investigate on the biological effects of the enhanced NAD metabolism in psoriatic skin through the supplementation of NAM to cultures of epidermal keratinocytes, fibroblasts and dermal endothelial cells activated by inflammatory psoriasis-related cytokines. Our results lead to identify the NAMPT-mediated NAD salvage pathway as the key metabolic pathway involved in the accumulation of intracellular NAD in psoriatic skin lesions, and to confer to it a pathogenic role in psoriasis skin. 

## 2. Materials and Methods

### 2.1. Human Subjects

Twenty-five patients (aged 40–70 years) with mild-to-severe chronic plaque psoriasis (Psoriasis area and severity index: 4–45) were included in this study. Biopsies were taken from skin plaques in both LS and nonlesional (NLS) (3-cm distant from the developing plaque) areas, all from the same psoriatic patients. In parallel, skin biopsies were also taken from 10 healthy volunteers undergoing plastic surgery. All these skin biopsies were analyzed for the intracellular NAD content, as below described (NAD^+^ measurement paragraph). Among psoriasis patients, *n* = 6 received subcutaneous injections of 300 mg Secukinumab (Cosentyx, Novartis) once a week for 2 months, after an induction phase. Biopsies were taken from skin plaques at sites overlapping LS before treatment (week 0) and after 8-week treatment and at NLS, 3-cm distant from the evolving plaques. These skin biopsies were subjected to Real Time (RT)-PCR analysis. 

This study was approved by the Ethical Committee of the IDI-IRCCS Hospital, Rome (Prot. N. 474/1/2016; study: “Studio delle chinurenine in pazienti affetti da psoriasi”) and performed accordingly to the Declaration of Helsinki. Informed consent was signed by all study subjects.

### 2.2. Cell Cultures and Treatments

Human keratinocytes were established from sun-protected skin of healthy individuals (*n* = 3 strains) and cultured as previously reported [35,36]. Cells were seeded (1.2–2 × 10^4^/cm^2^) on a feeder layer of irradiated 3T3 fibroblasts and cultured as previously described [37]. Second- or third-passage keratinocytes were used in all experiments, with cells cultured in the serum-free keratinocyte growth medium (KGM, Clonetics, San Diego, CA, USA), for at least 3–5 days (at 60–80% confluence) before performing treatments. Some experiments were performed on keratinocyte cultures undergoing terminal differentiation, achieved by growing cells at 100% of confluence (t0) and, thus, keeping them in culture for additional 4 days. 

Fibroblast cultures obtained from the dermis of skin biopsies (*n* = 3) were growth in DMEM (Lonza, Basel, Switzerland) supplemented with 10% FBS, 4 mM glutamine, 100 U/mL penicillin, and 100 mg/mL streptomycin (all from Lonza). Second- or third-passage fibroblasts were used in all experiments [38]. 

HDMECs were isolated from foreskin of donors as previously described [39] and growth in endothelial cell growth medium (EGM, Lonza). A pool of HDMECs derived from four different healthy donors was used at passages from 2 to 4. Endothelial basal medium (EBM, Lonza) was used as the starvation medium.

Stimulations of keratinocytes and fibroblasts with 200 U/mL recombinant human (rh) IFN-γ and 50 ng/mL of *rh*TNF-α were performed in keratinocyte basal medium (KBM, Clonetics) or serum-deprived DMEM, used as starvation medium, respectively. HDMECs were activated with the only *rh*TNF-α (10 ng/mL).

*rh*NAMPT (6424-VF, R&D SystemS, Minneapolis, MIN, USA) was used at a 10–500 ng/mL dose range. NAM was provided from IDI Farmaceutici s.r.l. (Pomezia, Italy) and administered at 0.05–1.5 mM dose range in presence or absence of cytokines. In some experiments, FK866 (IC50 0.09 nM, Sigma Aldrich, St-Louis, MO, USA) was added to cultures and used at 0.1 µM concentration. Cytotoxicity of NAM and FK866 was previously tested by measuring the activity of lactate dehydrogenase (LDH) released from keratinocyte cultures or fibroblasts, using Cytotoxicity Detection Kit Plus-LDH (Roche Diagnostics, Milan, Italy), following the manufacturer’ instructions.

### 2.3. Immunohistochemistry

Paraffin-embedded sections were obtained from biopsies of psoriatic skin (*n* = 6) including LS at baseline (week 0) and after 8-week Cosentyx treatment, and adjacent NLS zones, as well as of healthy skin. All sections were counterstained with Mayer’s Hematoxylin and eosin and photographed in five adjacent fields at a magnification × 100.

### 2.4. NAD Measurement 

NAD extraction from skin tissues and cell cultures and subsequent quantitative analyses were performed using the colorimetric NAD/NADH Assay Kit (Ab65348, AbCam, Cambridge, UK). NAD^+^ and NADH content was expressed as ng/mg or pg/µg of total proteins. 

### 2.5. RNA Isolation and RT-PCR

Total RNA was extracted from human skin biopsies by using RNeasy Lipid Tissue Kit (Qiagen, Chatsworth, CA, USA). Total RNA was extracted from keratinocyte, fibroblast or endothelial cultures stimulated with IFN-γ/TNF-α in presence or absence of NAM or FK866 by using the TRIzol reagent (Invitrogen, Carlsbad, CA, USA). 

mRNA was reverse-transcribed into complementary DNA by using SuperScript IV VILO reaction master mix (Invitrogen) and analyzed by real-time PCR. HPRT1 was used as housekeeping gene, as reported in the Figure legends. Primer pairs used in PCR reactions are listed in the table reported in Table 1. The probe for QPRT detection was provided by Applied Biosystems (Hs00204757 M1). Fluorescence intensity was analyzed by Quant Studio 5 (Thermo Fisher, Waltham, MS, USA), using SYBR Green PCR reagents or Taqman PCR Master Mix. Values obtained from triplicate experiments were averaged, and data are presented as means of 2^-DDCT values ± SD.

### 2.6. Immunoblotting and Densitometry

Protein extract preparation and immunoblotting were performed according to standard procedures [40,41]. The Abs used for the study are as follows: anti-ΔNp63 (Santa Cruz Biotechnology, Santa Cruz, CA, USA), -cyclin D (Santa Cruz Biotechnology), -pRb (Cell Signalling Technologies, Denvers, MA, USA), -Rb (Santa Cruz Biotechnology), anti-keratin (KRT)10, anti-loricrin (both from Covance, Meryville, CA, USA), and anti-β-actin (all from Santa Cruz Biotechnology, Santa Cruz, CA, USA). Filters were properly developed with anti-mouse, anti-goat, or anti-rabbit Ig Abs conjugated to HRP using the ECL-plus detection system (GE Healthcare, Chicago, IL, USA) or, otherwise, the SuperSignal West Femto kit (Pierce, Rockford, IL, USA) by using a ChemiDoc MP Imaging System (Bio-Rad, Hercules, CA, USA) supported by the Molecular Analyst Image software (https://imagej.nih.gov/ij/). Band intensities were evaluated in three independent experiments and reported as means of densitometric intensity (D.I.) ± SD. 

### 2.7. Proliferation Assays 

Proliferation of keratinocytes, fibroblasts, or endothelial cells was evaluated by using CyQuant Cell proliferation Kit (ThermoFisher Scientific), which measures the total DNA content. Briefly, 0.5–1 × 10^4^ cells were grown for 24, 48, and 72 h in 96-well plates in starvation medium and, after specific treatment, stained with CyQUANT dye, whose emission fluorescence was measured at 530 nm in EnSight multimode plate reader (Perkin Elmer, Waltham, MC, USA). Alternatively, 5−8 × 10^4^ keratinocytes, fibroblasts, or endothelial cells were seeded in 12-well plates and, the day after, starved. After specific treatments, viable cells were evaluated by trypan blue exclusion test.

In selected experiments, HDMECs were treated with fibroblast- or keratinocyte- conditioned medium. Briefly, fibroblasts or keratinocytes were seeded in 6-well plate and, after reaching about 70% confluence, cells were stimulated for 3 h with IFN-γ (200 U/mL) and TNF−α (50 ng/mL) in serum-deprived DMEM or KBM, respectively. Medium with stimuli was removed and basal medium was added for 24 h. Next, HDMECs were seeded in 96-well plates in EGM and 1 day after cells were starved and treated with conditioned medium of fibroblasts or keratinocytes, diluted 1:1 (*v*/*v*) in EBM, in the presence or not of a commercial anti-NAMPT neutralizing Ab (0.5 µg/mL) (ab236874, Abcam, Cambridge, United Kingdom) or a control IgG ab (0.5 µg/mL) (SC-2027, Santa Cruz Biotechnology). After 24 h of stimulation, the number of viable cells was determined by CyQUANT Cell Proliferation Assay.

### 2.8. Wounding Migration Assay

At 90% confluence, the endothelial monolayers in 60 mm culture dishes were wounded with a sterile p-200 pipette. Plates were rinsed with serum-free medium to remove cellular debris, and fresh medium containing NAMPT (100 ng/mL) or VEGF-A (50 ng/mL) was added. HDMECs were left to migrate for 36 h. Microscopy pictures were taken with a digital camera at different time points following treatments. The residual gap between migrating HDMECs was measured with a computer-assisted image analysis system (Axiovision; Zeiss, Oberkochen, Germany), and expressed as percentage of the initial scratched area. 

### 2.9. Flow Cytometry Analysis

Keratinocyte and fibroblast membrane expression of ICAM-1 and major histocompatibility complex (MHC) class II was evaluated using APC-conjugated anti-CD54 (clone 84H10; Immunotech, Marseille, France) and anti-HLA-DR (clone L243, BD Pharmingen, Franklin Lakes, NJ, USA) monoclonal Abs, respectively, whereas MHC-class I expression on keratinocyte membrane was detected by using APC-conjugated anti-human MHC-class I (clone 51-10C9, BD Pharmingen). Cells were analyzed by Accuri C6 Flow cytometer (BD) equipped with Cell Quest software (BD).

### 2.10. Enzyme-Linked Immunosorbent Assays (ELISA)

CCL20 and eNAMPT were measured using Duoset kits (R&D Systems), whereas CCL5, CCL2, and CXCL8 levels were measured with OptEIA^TM^ kits (BD Pharmingen, Milan, Italy), in cell-free supernatants (sups) from resting or stimulated keratinocyte, fibroblast and/or endothelial cultures, according to the manufacturer’s protocols. The plates were analyzed in an ELISA reader mod.3550 UV BioRad. Results are graphed as pg or ng/10^6^ cells ± SD. 

### 2.11. Statistical Analysis

Differences between groups were evaluated by the Mann–Whitney U or paired Student’s t test, as specified in the figure legends, by using GraphPad prism Software (La Jolla, CA, USA). Significance was assumed at a *p* value of 0.05 or less.

## 3. Results

### 3.1. NAD Content Is Enhanced in LS Psoriatic Skin and Is Associated to High NAMPT Transcriptional Levels

**Figure 1:**
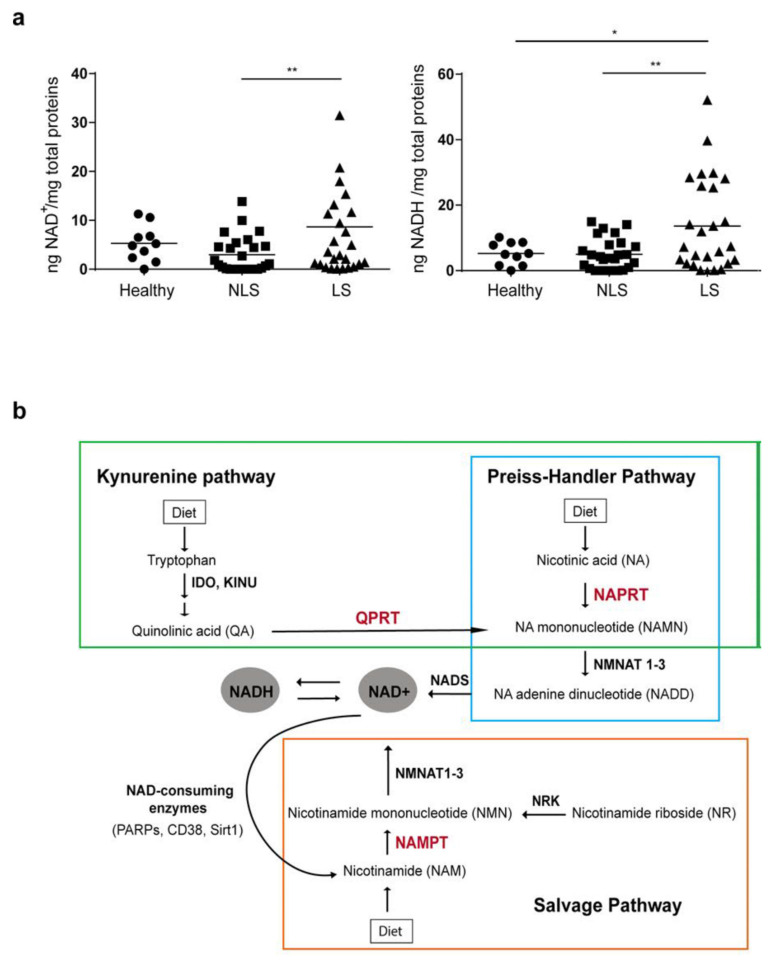
Intracellular NAD pool is higher in lesional (LS) skin of psoriasis patients than in uninvolved, non-lesional (NLS) skin. (**a**) NAD^+^ and NADH levels were quantified by colorimetric assay in NLS and LS skin specimens of target plaques obtained from patients (*n* = 25) affected by moderate-to-severe plaque psoriasis, as well as in healthy biopsies from donors (*n* = 10). The results are shown as individual values (ng/mg total proteins) and mean. Statistical significance was assessed by Mann–Whitney U test. * *p* ≤ 0.05 and ** *p* ≤ 0.01. (**b**) Graphical representation of the NAD biosynthetic pathways. NAD can be synthetized de novo starting from tryptophan (KYNUrenine pathway, green rectangle), or through salvage routes from NAM or NR (salvage pathway, orange rectangle), or metabolizing NA in the Preiss–Handler pathway (light blue rectangle). The rate-limiting enzymes of each biosynthetic pathway are indicated in red, the other enzymes involved in the reactions in black. NAD synthetized from NAM via NAMPT is in turn used by NAD-consuming enzymes which release NAM, making it available for continuous NAD regeneration.

NAD can exist as NAD^+^ and NADH, with the redox couple NAD^+^/NADH participating in numerous reactions requiring electron exchanges, such as glycolysis, β-oxidation, citric acid cycle, oxidative phosphorylation, and pyruvate-to-acetyl-CoA interconversions [42]. Aimed at clarifying the controversial NAD content in psoriatic skin, NAD^+^ and NADH levels were evaluated by colorimetric assay in NLS and LS skin zones, obtained from patients (*n* = 25) affected by moderate-to-severe plaque psoriasis, as well as in healthy skin from donors (*n* = 10). As shown in Figure 1a, a significant increase of both NAD^+^ and NADH levels was observed in LS areas compared to those observed in uninvolved skin. For NADH levels, a significant difference was also observed between healthy and LS groups. In contrast, no significant difference was observed in NAD^+^ or NADH amount between healthy and NLS skin groups. As illustrated in Figure 1b, NAD can be synthetized from NAM, tryptophan, or NA via three distinct NAD synthesis pathways, named salvage, kynurenine, and Preiss-Handler pathways, respectively. Salvage and Preiss-Handler pathways share NMNAT1-3 enzymes, which catalyze the final key steps of NAD synthesis. 

Aimed at overviewing the expression of key enzymes involved in NAD synthesis pathways in psoriasis skin context, two RNA-seq datasets (GSE13355 and GSE41662) were analyzed for differentially expressed genes among healthy control, NLS and LS psoriatic skin. 

**Figure 2:**
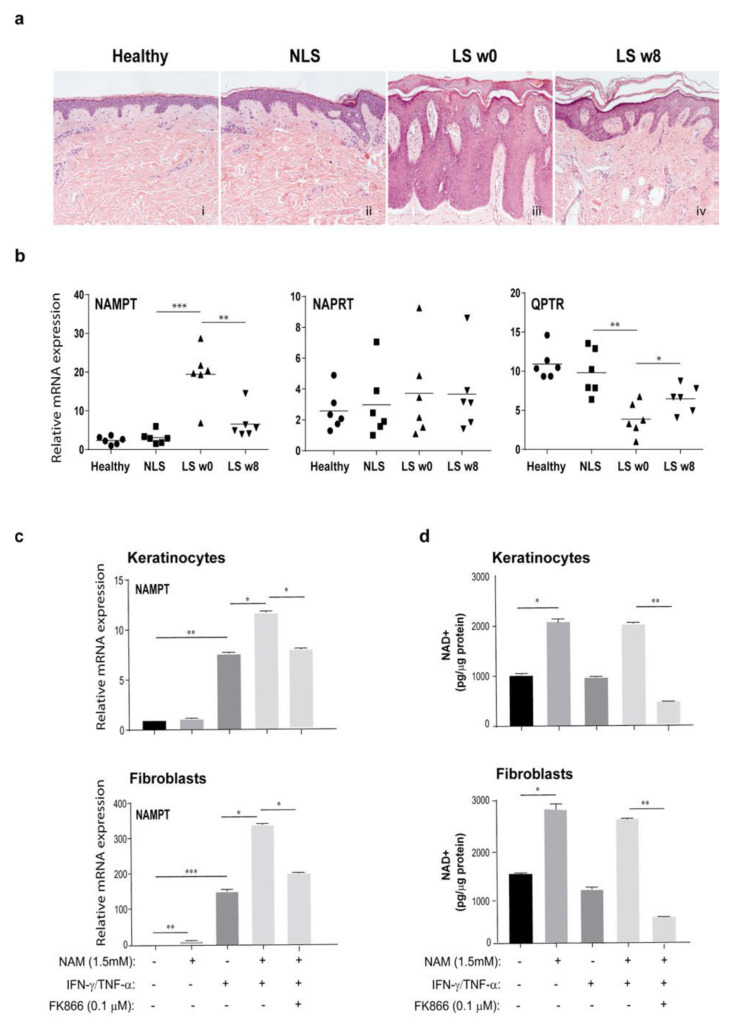
NAMPT mRNA levels are up-regulated in vivo in skin of psoriatic patients and induced in vitro in keratinocytes and dermal fibroblasts by combination of IFN-γ and TNF-α with ΝAΜ. (**a**) The histopathological features of psoriasis were examined in psoriatic skin (*n* = 6), including non-lesional (NLS, ii), lesional (LS) zones of evolving plaques, before (week 0, w0) (iii) and after 8 weeks (w8) (iv) of secukinumab treatment, counterstained with Mayer’s hematoxylin and eosin (H&E). One out of six representative stainings of psoriatic skin biopsies are shown. Specimens from healthy skin was also included (i). Bars, 100 μm. (**b**) mRNA expression of NAMPT, NAPRT, and QPRT were analyzed by RT-PCR on healthy, NLS, LS w0, and LS w8 biopsies (*n* = 6) and normalized to HPRT1 levels. Statistical significance was assessed by Mann–Whitney U test, * *p* ≤ 0.05, ** *p* ≤ 0.01, and *** *p* ≤ 0.001. (**c**,**d**) Keratinocyte and fibroblast cultures were left untreated or treated with a combination of IFN-γ/TNF-α in presence or absence of NAM (1.5 mM) for 24 h. Keratinocytes and fibroblasts activated by cytokines in presence of NAM were concomitantly exposed to FK866 (0.1 µM). (**c**) NAMPT mRNA expression was analyzed by RT-PCR and normalized to HPRT1 levels. (**d**) NAD^+^ levels were quantified by colorimetric assay and expressed as mean of pg/µg of total proteins. All data shown are the mean of three different experiments ± SD. Statistical significance was assessed by paired Student’s *t* test, * *p* ≤ 0.05, ** *p* ≤ 0.01, and *** *p* ≤ 0.001.

As shown in Figure S1 (panel a), IDO-1 and KYNU enzymes, involved in the first two steps of the kynurenine pathway, were significantly over-expressed in LS skin of psoriatic patients, compared to NLS and healthy skin samples. These results fit with a previous study reporting the upregulation of these two metabolic enzymes in psoriatic lesions [43]. Viceversa, transcriptional levels of QPRT, the enzyme involved in the synthesis of NAD by quinolinic acid, were significantly reduced in LS skin of psoriatic patients (Figure 2c). 

The transcriptional expression of NAMPT, the enzyme of the salvage pathway converting NAM in NMN, strongly increased in LS samples compared to healthy or NLS skin, whereas mRNA levels of NAPRT, an enzyme of the Preiss-Handler pathway converting NA in NAMN, remain almost constant among groups (Figure S1, panel b). In contrast, the levels of NMNAT2 and NMNAT3, two enzymes shared by Preiss-Handler and salvage pathways, slightly decreased in LS samples compared to healthy and NLS groups, whereas NMNAT1 expression remained similar among groups (Figure S1, panel c). Of note, in line with previous reports, SIRT1 enzyme was found to be down-regulated in LS compared to NLS group [44,45] whereas CD38 was upregulated (data not shown).

To validate the expression of the key enzymes of NAD metabolic pathways in psoriasis lesions, we collected skin samples from patients affected by plaque psoriasis and undergone to therapeutic treatment with the anti-IL-17A antibody, secukinumab. We first examined the histopathological features of psoriasis LS before (w0) and after therapy (w8), as well as of NLS and healthy tissues by H&E staining. As shown in Figure 2a, hyperkeratosis with parakeratosis, dilated dermal blood vessels, and inflammatory infiltration were evident in psoriasis lesions. In skin biopsies taken 8 weeks after therapy with secukinumab from the same target plaques analyzed at baseline, most of the histological hallmarks of psoriasis ameliorated (Figure 2a). Of note, NAMPT expression was strongly upregulated in LS samples, and it was reduced after therapy, becoming quite similar to that observed in healthy or NLS skin (Figure 2b). In parallel, NAPRT expression was similar among the experimental groups and its expression was not influenced by anti-IL-17A treatment (Figure 2b). Viceversa, QPRT expression was significantly downregulated in psoriasis lesions at baseline compared to QPRT observed in healthy or NLS groups, and it was restored by secukinumab treatment (Figure 2b). 

### 3.2. NAM Metabolism Synergizes with Psoriatic Cytokine Milieu in the Upregulation of NAMPT Expression

To investigate whether inflammatory cytokines could regulate the expression of the metabolic enzymes QPRT, NAPRT, and NAMPT, their transcriptional expression was analyzed in keratinocyte and fibroblast cultures stimulated with a combination of IFN-γ and TNF-α for 18 h. As shown in Figure 2c, NAMPT mRNA expression was strongly induced in both cell groups by cytokines, which acted synergistically. IFN-γ alone significantly induced NAMPT, whereas it was only moderately upregulated by TNF−α (data not shown). In contrast, QPRT and NAPRT mRNA expression was not influenced by cytokines in keratinocyte cultures, whereas NAPRT mRNA levels were downregulated by treatments in fibroblasts (Figure S2). QPRT transcriptional expression was not affected by the combination of IFN-γ and TNF-α in fibroblasts (Figure S2). IL-17A alone had no effect on QPRT, NAPRT, and NAMPT expression in any cell types, even if it synergizes with IFN-γ and TNF-α in NAMPT induction (data not shown). 

In order to investigate whether NAM, a precursor of NAD^+^, could affect the expression of the metabolic NAD enzymes themselves, we incubated keratinocyte and fibroblast cultures with NAM (1.5 mM) in presence or absence of IFN-γ and TNF-α for 18 h. Interestingly, as shown in Figure 2c, NAM administration synergized with IFN-γ/TNF−α in the induction of NAMPT expression in both keratinocyte and fibroblast groups. These results were consistent with the 2-fold increase of the intracellular NAD^+^ levels observed in both untreated and IFN-γ/TNF-α−treated cell types following NAM administration (Figure 2d). To confirm that this effect strictly depended on intracellular NAD^+^ levels, keratinocytes and fibroblasts, activated by IFN-γ/TNF-α and treated with NAM, were also exposed to FK866 (0.1 µM), a highly specific non-competitive inhibitor of NAMPT, which prevents the conversion of NAM into NAD^+^ through the salvage pathway [46]. As shown in Figure 2c, FK866 significantly reduced the NAMPT levels in IFN-γ/TNF-α-treated keratinocytes and fibroblasts, and this effect was consistent with a NAD^+^ drop observed in both untreated or IFN-γ/TNF-α-treated cell types exposed to FK866 (Figure 2d). In contrast to NAMPT, NAPRT expression was not regulated by intracellular NAD^+^ boosting in keratinocytes and fibroblasts, whereas QPRT mRNA levels were reduced by NAM administration solely in keratinocyte cultures (Figure S2).

Taken together, our data further confirm the microarray expression profile results, thus suggesting that the abnormal upregulation of NAMPT in psoriatic lesions, due at least in part to the inflammatory cytokine milieu and NAD metabolism itself, contributes to psoriasis pathogenesis. 

### 3.3. Opposite Effects of NAM on Cytokine-Activated Human Keratinocytes and Dermal Fibroblasts 

**Figure 3:**
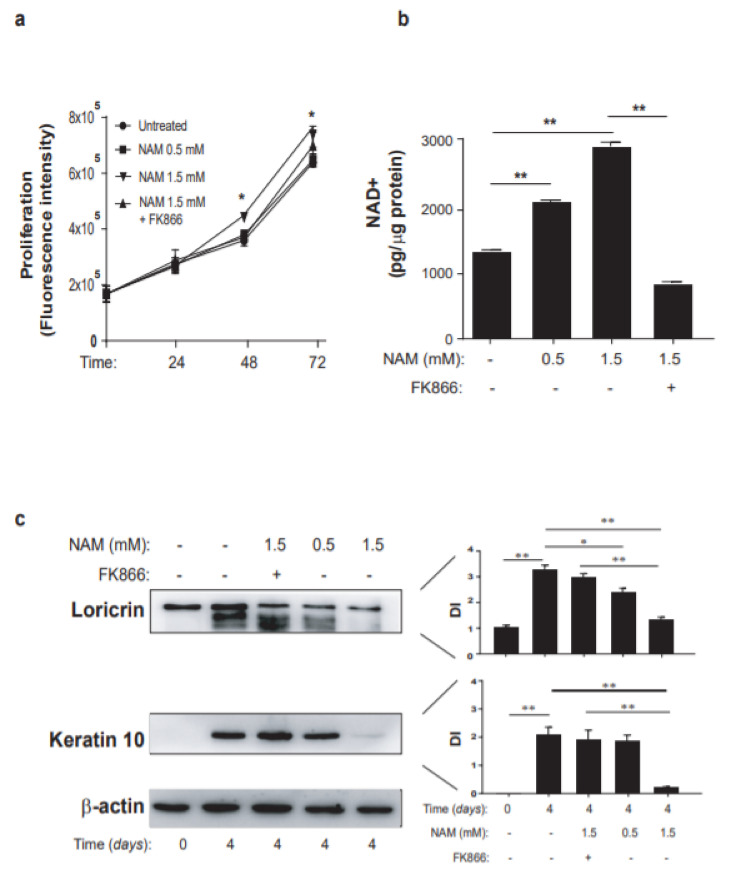
NAM induces proliferation and prevents differentiation of human primary keratinocytes. (**a**) Cell proliferation was evaluated by CyQUANT assay performed on keratinocyte cultures growth in KBM and left untreated or treated with increasing doses of NAM (0.5–1.5 mM), in presence or not of FK866 (0.1 µM) for 24, 48, and 72 h. Data are shown as the mean of fluorescence intensity values obtained from three independent experiments carried out on *n* = 3 different strains ± SD. * *p* ≤ 0.05 by paired Student’s *t* test. (**b**) Keratinocyte cultures were treated with increasing doses of NAM (0.5–1.5 mM) in KBM for 24 h or left untreated. NAD^+^ levels were quantified by colorimetric assay and expressed as mean of pg/µg of total proteins. Data are expressed as mean of three independent experiments ± SD. ** *p* ≤ 0.01 by paired Student’s *t* test. (**c**) Keratinocyte cultures were subjected to culture conditions determining terminal differentiation. The latter was achieved by growing cells at 100% of confluence (t0) and, thus, keeping them in culture for another 4 days in presence or absence of increasing NAM doses. Where indicated, cells were exposed treated with FK866 (0.1 µM) together with NAM. Loricrin and keratin 10 protein levels were analyzed by WB. DI indicates the densitometric intensity of the indicated proteins normalized for β-actin shown in one representative of three different WB. Data are represented as mean ± SD. * *p* ≤ 0.05 and ** *p* ≤ 0.01 assessed by paired Student’s *t* test.

In order to investigate the implication of NAD metabolism in the pathogenic processes occurring in psoriasis, keratinocytes and dermal fibroblasts activated by pro-inflammatory cytokines were exposed to NAM and analyzed in terms of expression of inflammatory genes and differentiation/proliferation. We found that keratinocyte cultures exposed to increasing doses of NAM showed an enhanced proliferation compared to control cells, with a significant difference at 48-h and 72-h treatment with NAM at the higher dose (1.5 mM) (Figure 3a). This induction was mediated by intracellular NAMPT, since the presence of FK866 reduced the proliferation induced by NAM (Figure 3a). Moreover, it was consistent with the accumulation of the intracellular NAD, which increased in a dose-response manner upon NAM exposure for 24 h and dropped in presence of FK866 (Figure 3b). At molecular level, NAM administration resulted in increased levels of proteins related to cell cycle, such as cyclin D and phosphorylated Rb, as well as to proliferation, including ΔNp63 (Figure S3, panel a).

As previously reported [47], IFN-γ decreases proliferation of keratinocyte cultures whereas TNF-α induces apoptosis [48]. In order to investigate whether NAM could contrast the effects of the inflammatory IFN-γ and TNF-α cytokines on keratinocyte viability and proliferation, cells were treated with NAM and stimulated with IFN-γ and TNF-α for 48 h. As shown in Figure S3, NAM (1.5 mM) was not able to revert the IFN-γ/TNF-α-induced inhibition of proliferation (panel b). 

Next, we analyzed the effects of NAM on keratinocyte differentiation. In line with recent data of Tan et al. [49], keratinocyte cultures undergone differentiation in presence of NAM showed reduced levels of loricrin and keratin 10 in a dose-response manner, compared to the untreated control cells, thus confirming the prevention of NAM in keratinocyte differentiation (Figure 3c). Of note, the expression of differentiation markers was restored by treatment of cells with FK866 (Figure 3c), demonstrating the involvement of the intracellular NAMPT in impairing differentiation determined by NAM. 

**Figure 4:**
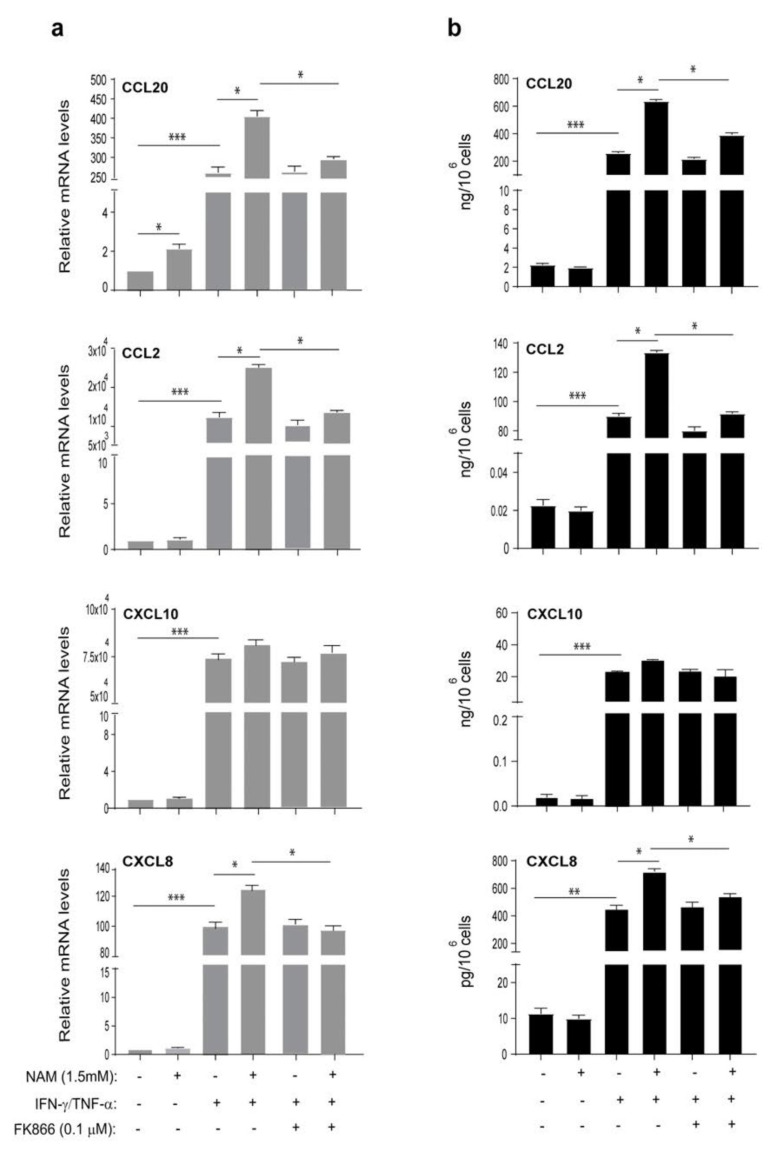
Intracellular NAD^+^ boosting by NAM supplementation enhances IFN-γ/TNF-α-induced expression and release of CCL20, CCL2 and CXCL8 by human keratinocytes. (**a**,**b**) Keratinocyte and fibroblast cultures were left untreated or stimulated with a combination of IFN-γ (200 U/mL)/TNF-α (50 ng/mL) in presence or absence of NAM (1.5 mM) for 24 h. Keratinocytes and fibroblasts activated by cytokines in presence of NAM were concomitantly treated with FK866 (0.1 µM). (**a**) CCL20, CCL2, CXCL10 and CXCL8 mRNA expression was detected by real-time PCR and normalized to HPRT1 levels. (**b**) Chemokine production was analyzed by ELISA in supernatants obtained by cell cultures treated as described above. All data shown are the mean of three different experiments ± SD. * *p* ≤ 0.05, ** *p* ≤ 0.01, and *** *p* ≤ 0.001 compared with the untreated cultures, as assessed by paired Student’s *t* test.

We finally evaluated whether NAM could modulate the expression of a variety of molecules involved in the induction or control of skin inflammation in activated keratinocytes. As shown in Figure 4, NAM significantly synergized with IFN-γ and TNF-α in the induction of CCL20, CCL2, and CXCL8 mRNA expression (panel a) and release (panel b), whereas it did not influence IFN-γ/TNF-α-induced expression and production of CXCL10 (Figure 4a,b). Similarly, ICAM-1, HLA-DR, and MHC class I induction by IFN-γ/TNF-α was not affected by NAM administration (Figure S3c). 

**Figure 5:**
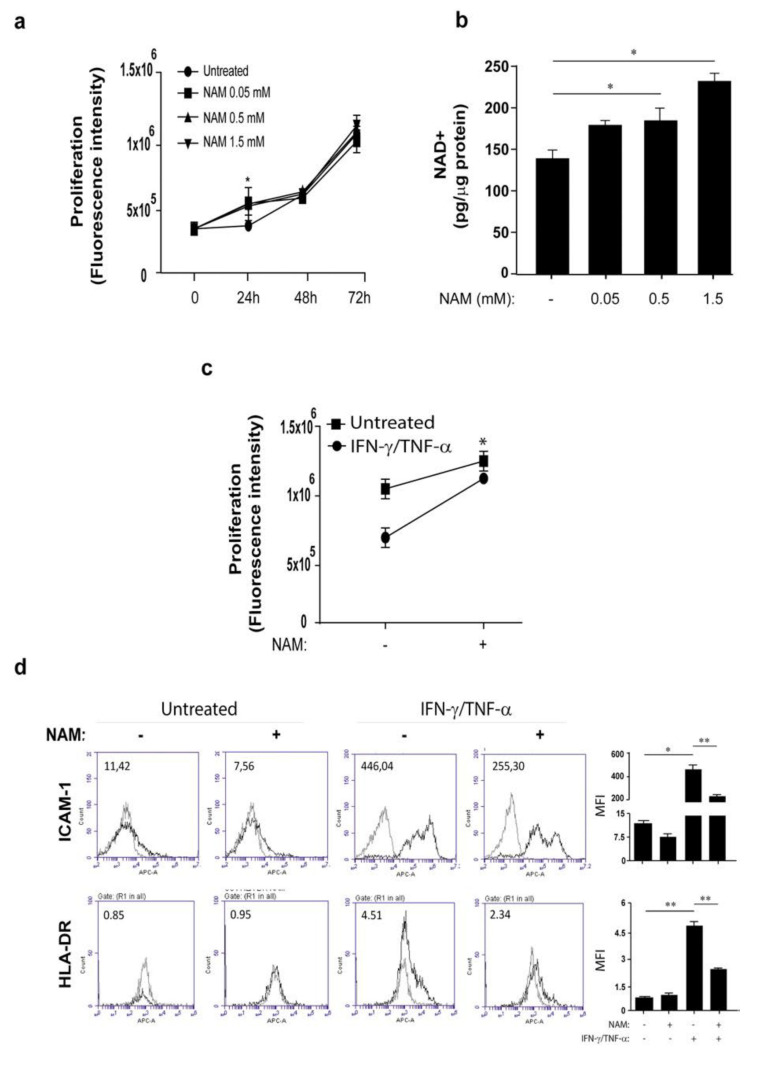
NAM induces proliferation of human fibroblasts and reduces IFN-γ/TNF-α-induced adhesion membrane molecule expression. (**a**) Cell proliferation was evaluated by CyQUANT assay on primary cultures of human fibroblasts in DMEM deprived of serum and left untreated or treated with increasing doses of NAM (0.05–1.5 mM) for 24, 48, and 72 h. (**b**) NAD^+^ levels were quantified in fibroblast cultures treated with increasing NAM doses by colorimetric assay and expressed as mean of pg/µg of total proteins. (**c**) Cell proliferation was evaluated in fibroblasts left untreated or stimulated with IFN-γ (200 U/mL)/TNF-α (50 ng/mL) in presence or absence of NAM (1.5 mM) for 24 h. In (**a**,**c**), data are shown as the mean of fluorescence intensity values obtained from three independent experiments ± SD carried out on *n* = 3 different strains. In (**b**) NAD^+^ levels were quantified by colorimetric assay and expressed as mean of pg/µg of total proteins. All data shown are the mean of three different experiments ± SD. In (**a**–**c**), * *p* ≤ 0.05 by paired Student’s *t* test. (**d**) ICAM-1 and HLA-DR expression was evaluated by flow cytometry analysis on fibroblasts stimulated for 24 h by IFN-γ/TNF-α and treated with NAM (1.5 mM). Data are shown as means of fluorescence intensity (MFI). All data shown are the mean of three different experiments ± SD. * *p* ≤ 0.05 and ** *p* ≤ 0.01, as assessed by paired Student’s *t* test.

In dermal fibroblasts, NAM was converted in intracellular NAD^+^ in a dose-response manner and determined an increase of proliferation at 24 h of treatment (Figure 5). Of note, when NAM was co-administered together with IFN-γ/TNF-α to fibroblast cultures, the effects of these cytokines on fibroblast viability were partially reverted, suggesting an its protective action (Figure 5c). In contrast to that observed in keratinocytes, NAM significantly counteracted ICAM-1 and HLA-DR membrane molecule expression induced by IFN-γ and TNF-α in fibroblasts (Figure 5d). Finally, NAM did not influence the expression and release of inflammatory cytokines and chemokines, except for CCL5, whose IFN-γ/TNF−α-induced expression was enhanced by NAM (data not shown). 

Taken together, these results suggest opposite effects of NAD metabolism on proliferation/differentiation and inflammatory programs executed by skin resident cells and typically involved in pathogenesis of psoriasis.

### 3.4. NAMPT Is Strongly Released by Activated Keratinocytes and Fibroblasts and Induces Proliferation and Inflammation in Dermal Endothelial Cells

**Figure 6:**
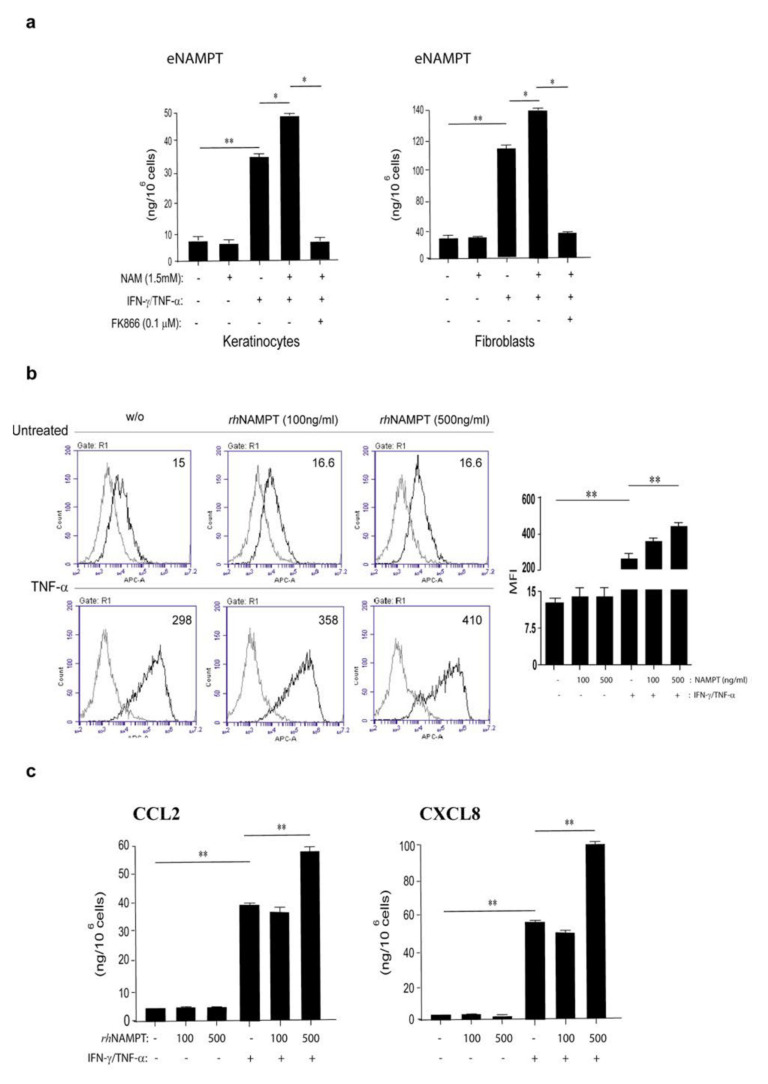
The release of extracellular NAMPT is strongly induced by pro-inflammatory cytokines and it is strictly regulated by intracellular NAD^+^ metabolism. (**a**) Extracellular NAMPT (eNAMPT) release was analyzed by ELISA in supernatants obtained by keratinocyte and fibroblasts left untreated or treated with a combination of IFN-γ/TNF-α in presence or absence of NAM (1.5 mM) for 24 h. When necessary, keratinocytes and fibroblasts activated by cytokines were concomitantly exposed to NAM and FK866 (0.1 µM) for 24 h. Data are expressed as mean of three independent experiments ± SD. * *p* ≤ 0.05 and ** *p* ≤ 0.01 by paired Student’s *t* test. (**b**) ICAM-1 expression was evaluated by flow cytometry analysis on HDMEC stimulated for 24 h with IFN-γ/TNF-α and treated or not with rh NAMPT at 100 ng/mL and 500 ng/mL. Data are shown as means of fluorescence intensity ± SD. ** *p* ≤ 0.01 by paired Student’s *t* test. (**c**) CCL2 and CXCL8 release was evaluated by ELISA on supernatants obtained from HDMEC cultures stimulated or not for 24 h with IFN-γ/TNF-α- in presence or not of rh NAMPT (100 ng/mL and 500 ng/mL). Data are expressed as mean of three independent experiments ± SD. ** *p* ≤ 0.01 was calculated by paired Student’s *t* test.

NAMPT is released by several kinds of cells, including pancreatic β-cells, myocytes, hepatocytes, and adipocytes [50]. In order to investigate whether skin resident cells could secrete NAMPT, cultures of keratinocytes and dermal fibroblasts were activated by IFN-γ and TNF-α, or left untreated, in presence or absence of NAM. As shown in Figure 6a, both activated keratinocytes and dermal fibroblasts released NAMPT, even though it was produced by fibroblasts at higher levels (~120 ng/10^6^ cells vs. 30 ng/10^6^). In line with transcriptional data reported in Figure 2d, NAM supplementation to cell cultures resulted in an increase of IFN-γ/TNF-α-induced NAMPT production (Figure 6a), whereas it did not influence NAMPT release in resting cultures. This up-regulation was strictly dependent on NAM to NAD^+^ conversion. Indeed, NAMPT inhibition by FK866 and consequent reduction of the intracellular NAD pool, determined a drastic drop in the release of NAMPT itself in IFN-γ/TNF-α-induced cells treated by NAM, which returned to levels similar to resting cells (Figure 6a).

Psoriatic skin is characterized not only by presence of immune cell infiltrate and keratinocyte hyperproliferation, but also by an increased dermal vascularity due to aberrant angiogenesis [51]. To this matter, previous studies reported that rh NAMPT promotes angiogenesis by inducing in vitro proliferation, migration and invasion of endothelial cells via MAPK and PI3K/AKT signaling pathways [52]. Furthermore, NAMPT induces the secretion of CCL2, VEGF, and MMP-2 production in human endothelial cells, which further enhance angiogenesis in auto-inflammatory loops [24,52].

In order to assess whether NAMPT had pro-angiogenic and pro-inflammatory effects in our settings, primary cultures of dermal endothelial cells were treated with two different doses of rh NAMPT and their proliferation was evaluated at 24 h and 48 h of treatment. As shown in Figure S4 (panel a), at both doses rh NAMPT induced HDMEC proliferation, which was significantly higher when compared to the untreated cells (growth in starvation medium), and quite similar to proliferation observed in cells growth in complete medium (EGM) at 24 h. Furthermore, in line with literature data [24,25,53], we found that NAMPT activated HDMEC migration by promoting wound healing 36h after scratching of HDMEC monolayer (Figure S4b). The wound closure in HDMEC culture exposed to rh NAMPT was similar to that observed in HDMEC treated by VEGF-A, a pro-angiogenic molecule (Figure S4b). Of note, we found that NAMPT significantly synergizes with TNF-α, the key cytokine trigger of endothelial cells inducing ICAM-1 membrane molecule (Figure 6b), whereas it did not influence the expression of VCAM or E-selectin (data not shown). Finally, differently from what was observed previously, NAMPT did not induce the production of CCL2 and CXCL8 chemokines in resting HDMEC cultures, even if it synergized with TNF-α in promoting their release (Figure 6c).

**Figure 7:**
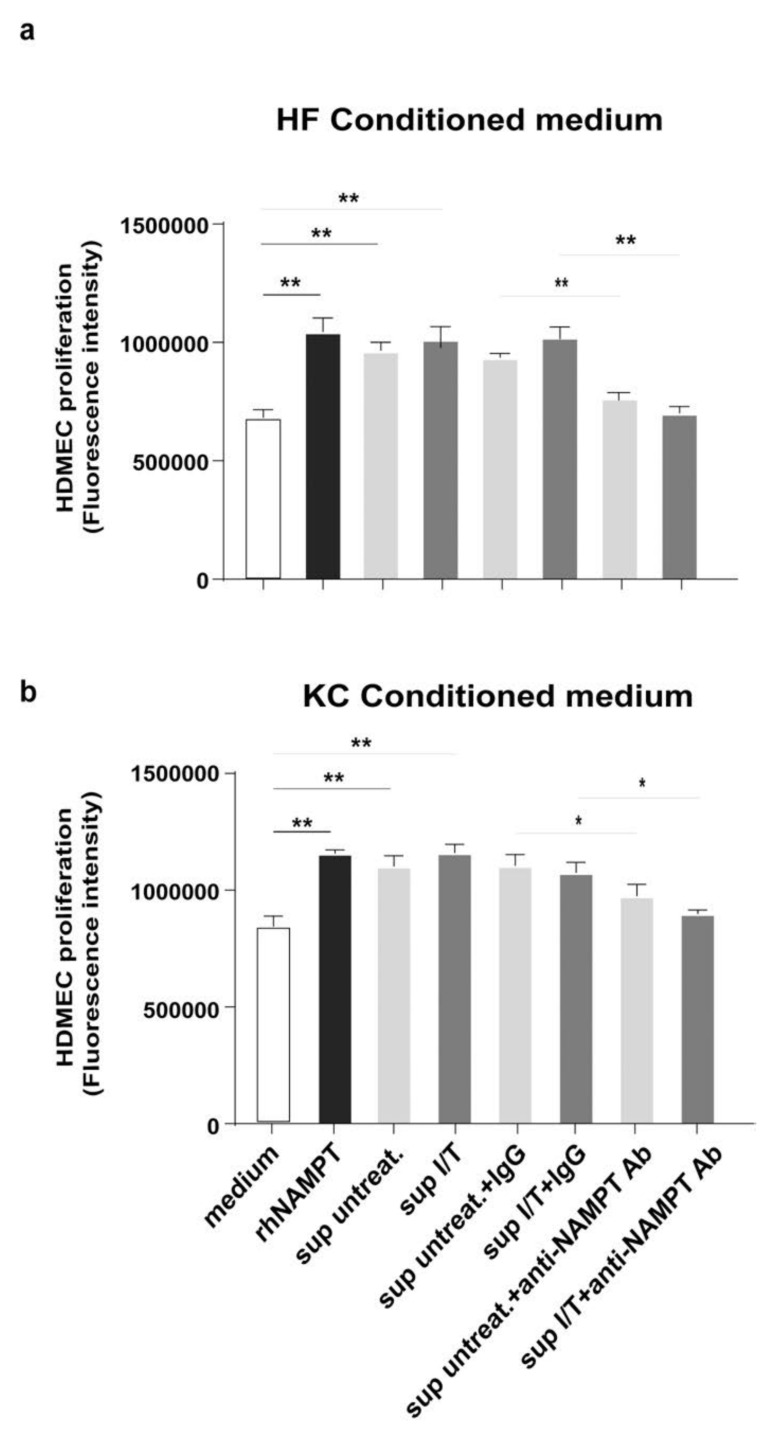
eNAMPT released by keratinocytes and fibroblasts induces HDMECs proliferation. (**a**,**b**) Cell proliferation was evaluated by CyQUANT assay performed on HDMECs stimulated or not for 24 h with sups of untreated or IFN-γ/TNF-α-treated fibroblasts (HF) (**a**) or keratinocytes (KC) (**b**), in presence or not of a NAMPT-neutralizing Ab, or a control IgG. In parallel, cells were stimulated with rh NAMPT (10 ng/mL), as positive control. Data are shown as mean of fluorescence intensity values obtained from three independent experiments ± SD. * *p* ≤ 0.05 and ** *p* ≤ 0.01 by paired Student’s *t* test.

To confirm that the extracellular NAMPT released from fibroblasts and keratinocytes was functionally active in inducing HDMEC proliferation, HDMECs were grown with culture medium conditioned by keratinocytes or fibroblasts treated or not with IFN-γ/TNF-α and pre-incubated or not with a commercial antibody (Ab) neutralizing NAMPT or with IgG, as negative control. In parallel, HDMECs were treated with recombinant NAMPT protein, as positive control. As shown in Figure 7, HDMEC stimulation with conditioned medium of both untreated or IFN-γ/TNF-α-treated fibroblasts (Figure 7a) and keratinocytes (Figure 7b) induced HDMEC proliferation compared to the medium, and cell proliferation was significantly reduced by anti-NAMPT Ab. Of note, HDMECs grown in presence of supernatants (sups) from activated (or not) fibroblasts (Figure 7a) and keratinocytes (Figure 7b), pre-incubated with an anti-NAMPT neutralizing Ab, showed a reduced proliferation, when compared to cells grown with sups incubated with a control IgG. These differences were more evident with medium conditioned by fibroblasts, in line with the higher release of eNAMPT observed in untreated and IFN-γ/TNF-α-treated sups of fibroblasts (untreated 23.3 ± 4.5 ng/10^6^ cells vs. treated 48 ± 2.5 ng/10^6^ cells) compared to those observed in sups of keratinocytes (untreated 4.6 ± 0.5 ng/10^6^ cells vs. treated 10.1 ± 1.5 ng/10^6^ cells). Similar results were obtained by neutralizing the extracellular NAMPT released by fibroblasts and keratinocytes with a newly generated monoclonal antibody (C269) able to neutralize the cytokine-like action of eNAMPT in vitro and in vivo [54]. These results confirm the pro-proliferative activity of the extracellular NAMPT released by skin resident cells on dermal endothelial cells.

## 4. Discussion

In this study, we describe the expression and potential inflammatory function of the NAD salvage pathway in psoriatic skin. To our knowledge, this is the first exploration on the role of this metabolic route in the pathogenesis of psoriasis.

Psoriasis is a chronic inflammatory skin disease arising from a complex interplay between hyper-proliferative keratinocytes and infiltrating immune cells. Th1/Th17 cells dominate the inflammatory immune infiltrate and activate auto-inflammatory loops sustained by cytokines and growth factors mainly released by keratinocytes and dermal fibroblasts [35,41]. Along with epidermal hyperplasia and inflammatory cell infiltration, prominent dermal vessel dilation is an important histological hallmark of psoriatic lesions. Here, dermal endothelial cells, activated by keratinocyte- and fibroblast-derived pro-angiogenic factors (i.e., VEGF and angiopoietins), express many adhesion molecules and promote leukocyte recruitment [55]. These factors, together with the inflammatory cytokine TNF-α, determine the de novo formation of vascular networks in psoriatic skin [55].

The interest addressed to NAD metabolism arises from our initial observation that skin lesions of patients affected by psoriasis are characterized by an enhanced intracellular NAD boosting, compared to uninvolved or healthy skin. In this manuscript, only NAMPT has been assessed, due to the fact that it is the only NAD salvage enzyme significantly upregulated in psoriatic LS skin. Of note, we found that NAMPT expression is strongly reduced by Secukinumab, an anti-IL-17A antibody successfully used in the treatment of psoriasis patients [56], suggesting its implication in disease pathogenesis.

NAD salvage is strictly coupled to NAD-consuming enzymes, such as sirtuins, PARPs, and CD38, which release NAM, which, in turn, supports NAD production via NAMPT-dependent salvage pathway. The dysregulation of this auto-regulatory loop could be also due to the abnormal expression of SIRTs in psoriasis, with the downregulation of SIRT1, SIRT2, SIRT3, SIRT4, and SIRT5 and upregulation of SIRT6 and SIRT7 in skin lesions skin, as previously reported [44,45].

However, NAD salvage is not the only NAD metabolic route to be altered in psoriatic skin. Indeed, a recent study demonstrated that the tryptophan metabolism enzymes, IDO-1 and KYNU, are also strongly expressed in psoriatic skin lesions, and their expression correlates with disease severity and inflammation. The upregulation of IDO-1 and KYNU results in the accumulation of metabolites downstream of KYNU, which have direct inflammatory effects on keratinocytes, endothelial cells, and activated T cells [43]. However, in this study we observed a decreased expression in QPRT, an enzyme downstream of KYNU, in psoriatic lesions, which could likely represent an attempt of epithelial and immune cells in counteracting the intracellular NAD+ boosting derived from NAD salvage.

**Figure 8:**
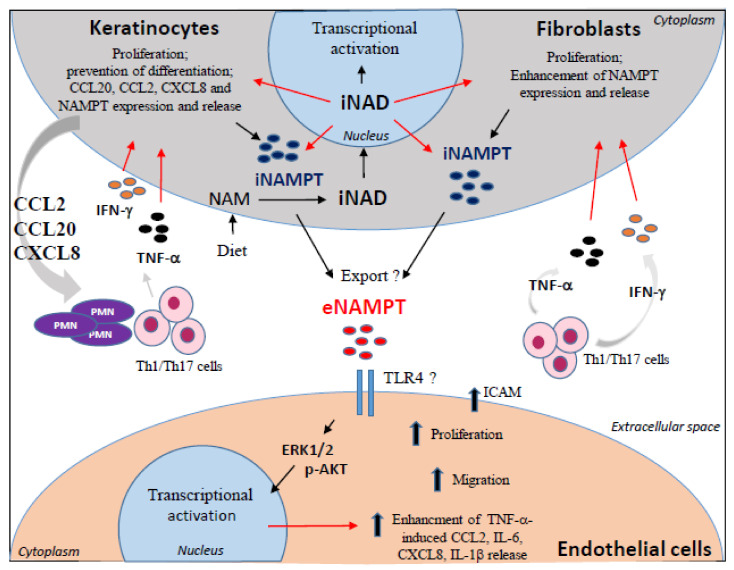
Model emphasizing the role of NAMPT-dependent NAD metabolism on the pathogenic crosstalk between keratinocytes, fibroblasts, and dermal endothelial cells in psoriatic skin. Psoriatic skin lesions are characterized by a high intracellular NAD+ boosting, compared to uninvolved skin. Accumulation of the intracellular NAD contributes to the enhancement of IFN-γ/TNF-α-induced release of inflammatory cytokines, such as CCL2, CCL20, and CXCL8, by keratinocytes, which fuel an auto-inflammatory loop by recruiting Th17/Th1 subpopulations and polymorphonuclear leukocytes (PMN). The high intracellular NAD levels also lead to keratinocyte hyperproliferation and impaired differentiation, thus contributing to the epidermal thickening typical of psoriatic skin lesions. Furthermore, upon exposure to IFN-γ and TNF-α cytokines, keratinocytes and dermal fibroblasts release high amounts of NAMPT, which is exported to the extracellular space by unknown mechanisms. The high intracellular NAD levels contribute to the induction of NAMPT expression in keratinocytes and fibroblasts. Keratinocyte- and fibroblast-released extracellular NAMPT (eNAMPT) acts on dermal endothelial cells in a paracrine fashion, by inducing their proliferation and migration, which contribute to a dysregulated angiogenesis. Finally, eNAMPT enhances TNF-α-induced expression of ICAM1 on membrane of endothelial cells, as well as their release of CCL2, CXCL8, and IL-6, which contribute to the extravasation and recruitment of inflammatory immune infiltrate into inflamed skin.

Based on our data, we propose a model for how NAD metabolism via salvage pathway may regulate the interplays between keratinocytes, fibroblasts, and endothelial cells in psoriatic skin (Figure 8). In support of this model, we provide evidence that both intracellular and extracellular forms of NAMPT contribute to the epithelial inflammation and high intracellular NAD levels (mimicked in vitro by NAM supplementation), determine impaired differentiation, and increased the proliferation of keratinocytes. We also found that these effects strictly depend on intracellular NAMPT, as demonstrated by using the allosteric inhibitor of NAMPT FK866 [57]. However, it cannot be excluded that the increased NAD levels are a consequence of the hyper-proliferation state of keratinocytes, and therefore an attempt to meet the energy demands of these cells.

For the first time, we show that the high intracellular NAD boosting, mediated by intracellular NAMPT, fuels an auto-inflammatory loop by enhancing the cytokine-dependent release of inflammatory chemokines by keratinocytes, in turn contributing to Th17/Th1 and neutrophil recruitment in inflamed skin (Figure 8). Of note, cytokines upregulated NAD metabolism by inducing the expression of NAMPT itself in both keratinocytes and dermal fibroblasts. These results are consistent with previous studies showing that NAMPT expression is induced by TNF-α, IL-6, IL-1β, or LPS in amniotic epithelial cells, macrophages, chondrocytes, and synovial fibroblasts [58,59]. In addition, a number of binding sites for the transcription factors STAT1/3, NF-κB, and AP-1/2, typically activated by cytokines and Toll-like receptor, have been identified in the proximal region of NAMPT promoter [60,61].

Interestingly, we found that intracellular NAMPT synergizes with inflammatory cytokines in transcriptionally inducing NAMPT itself in both keratinocytes and fibroblasts (Figure 8). This auto-regulatory mechanism depends on the NAD^+^-mediated action of transcription factors acting on NAMPT promoter [62]. Among them, the SIRT1-induced C/EBPα/β transcription factor could be potentially involved in NAMPT expression, as binding sites are present in NAMPT promoter [61]. Consistently, in CD34^+^ hematopoietic progenitor cells, intracellular NAMPT activates NAD^+^-dependent SIRT1, leading to the induction of C/EBPα/β, and, ultimately, to the upregulation of G-CSF and G-CSF receptor expression, fundamental for neutrophils differentiation. G-CSF, in turn, increases NAMPT levels in an auto-regulatory mechanism [63]. Further investigations will be needed to ascertain the potential involvement of NAD^+^-sirtuin-1-dependent pathway via C/EBPα/ϖ in the regulation of NAMPT expression in psoriasis.

Other than having intracellular functions, NAMPT is known to be secreted in the extracellular space by mechanisms unknown yet [64]. The most accredited hypothesis is that eNAMPT is exported through a “non-classical” secretory pathway, being its secretion not blocked by typical inhibitors of endoplasmic reticulum-Golgi secretory pathway [19,65].

Importantly, we found that keratinocytes and, at higher extent, dermal fibroblasts activated by inflammatory cytokines release high amounts of eNAMPT. NAMPT-dependent NAD salvage pathway seems to be important in the production of eNAMPT itself, since FK866 drastically reduces cytokine-induced NAMPT to basal levels in keratinocytes and fibroblasts. The mechanisms by which NAMPT-mediated NAD metabolism influence the extracellular export of NAMPT remained to be explored.

Based on our hypothesis, through a paracrine mechanism, keratinocyte- and fibroblast-released eNAMPT could directly act on dermal endothelial cells by inducing their proliferation and migration, and contributes to the dysregulated angiogenesis typically observed in psoriatic lesions (Figure 8). Finally, eNAMPT, by synergizing with TNF-α, could contribute to aberrant ICAM-1 expression in dermal endothelial cells, as well as CCL2, CXCL8, and IL-6 release—all molecules fundamental for the recruitment, retention, and activation of immune cells in inflamed skin (Figure 8).

Other than in epithelial compartment, NAMPT expression is also enhanced in peripheral blood mononuclear cells of patients with severe psoriasis [66], indicating its involvement in systemic inflammation mediated by circulating immune cells. Moreover, NAMPT is systemically elevated in patients with rheumatoid arthritis [67,68] or inflammatory bowel diseases [54]. Specific inhibitors or antibodies blocking NAMPT ameliorate the immune-mediated inflammation of experimental models of acute and chronic colitis and collagen-induced arthritis, by reducing intracellular NAD^+^ levels in inflammatory cells and circulating inflammatory cytokines [54,69,70]. The potential anti-inflammatory effects of intracellular NAMPT inhibitors and/or neutralizing NAMPT antibodies will be further investigated in experimental murine models of psoriasis.

In conclusion, this study proposes that NAMPT-mediated NAD salvage pathway contributes to psoriasis pathogenesis by amplifying epithelial auto-inflammatory responses in psoriasis. Although previous studies reported that NAD^+^-augmenting supplements improve skin aging [71] and has beneficial effects on skin appearance, for example reducing melasma [72], our results suggest that dietary NAD^+^-supplementation should be administrated with caution in skin disorders characterized by keratinocyte hyperproliferation and auto-inflammatory immune responses, such as psoriasis. Due to the implication of extracellular NAMPT in metabolic disorders and arthritis, circulating levels of NAMPT, as well as its potential inflammatory function in immune cells, in particular in Th17 lymphocytes, should be evaluated and further deepened also in terms of association with psoriasis co-morbidities.

## Figures and Tables

**Table 1:** Primers sequences used for real-time PCR.

**Gene**	**Forward**	**Reverse**
NAMPT	TGA ATG CCG TGA AAA GAA GA	AAT TTG TTG CCA CTG TGA TT
NAPRT	GTG AGG TGA ATG TCA TTGG	GGC CAC CAG CTT ATA GAC
CCL20	GTG CTG CTA CTC CAC CTC TG	TGT ATC CAA GAC AGC AGT CAAA
CCL2	CAC CAG CAG CAA GTG TCCC	CCA TGG AAT CCT GAA CCC AC
CXCL10	TGG CAT TCA AGG AGT ACC TCT CT	CTG ATG CAG GTA CAG CGT ACG
CXCL8	CTC TGT GTG AAG GTG CAG TTTT	GGG TGG AAA GGT TTG GAG TAT
HPRT1	ATG GAC AGG ACT GAA CGT CTT	CTT GAG CAC ACA GAG GGC TA

## Data Availability

Primary data: data access: GSE13355.

## Supplementary Materials

The following are available online at https://www.mdpi.com/article/10.3390/ijms22136860/s1.

## Abbreviations

NAM: nicotinamide; NMN, nicotinamide mononucleotide; IDO, Indoleamine-pyrrole 2,3-dioxygenase; KYNU, Kynureninase; QPRT, quinolinate phosphoribosiyltransferase; NAPRT, nicotinate phosphoribosyltransferase; NRK, nicotinamide riboside kinase; NAMPT, nicotinamide phosphoribosyltransferase; Sirt1, Sirtuin1; PARPs, poly ADP-ribose polymerases.
