# Supplementary material for: Enhanced NAMPT-Mediated NAD Salvage Pathway Contributes to Psoriasis Pathogenesis by Amplifying Epithelial Auto-Inflammatory Circuits
Source: Int J Mol Sci. 2021 Jun 25;22(13):6860. doi: 10.3390/ijms22136860 (PMC8267663; doi:10.3390/ijms22136860)
Supplement: Supplementary file 1 [file ijms-22-06860-s001.zip › ijms-1244041-supplementary.pdf]

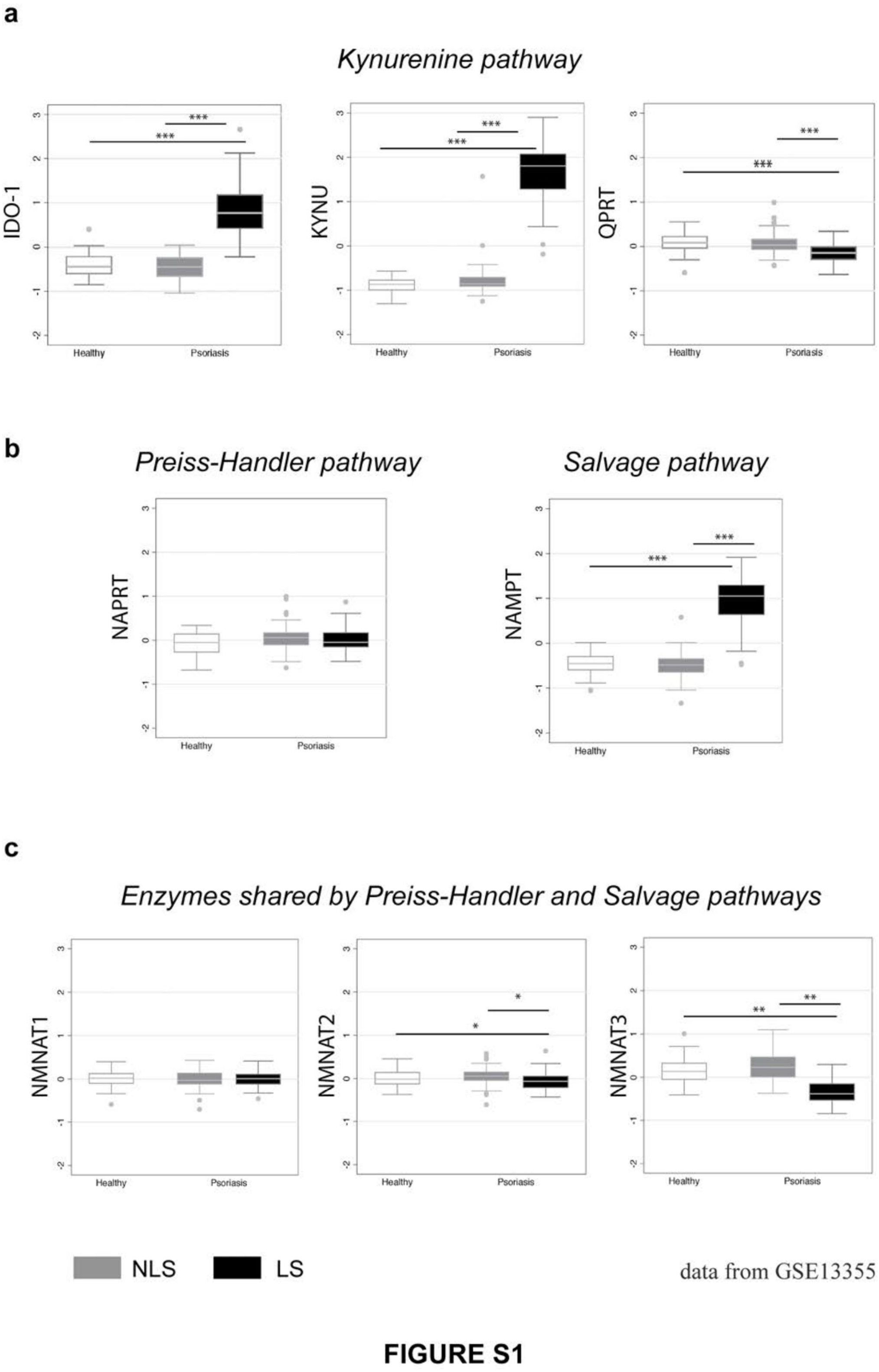

Figure S1. Expression data of the key enzymes involved in NAD synthesis pathways in healthy control, non-lesional (NLS) and lesional (LS) psoriatic skin tissues obtained from RNA-seq dataset

(GSE13355). Data are expressed as mean ± SD. *$p \leq 0.05$, ** $p \leq 0.01$ and ***$p \leq 0.001$ by paired Student's *t* test.

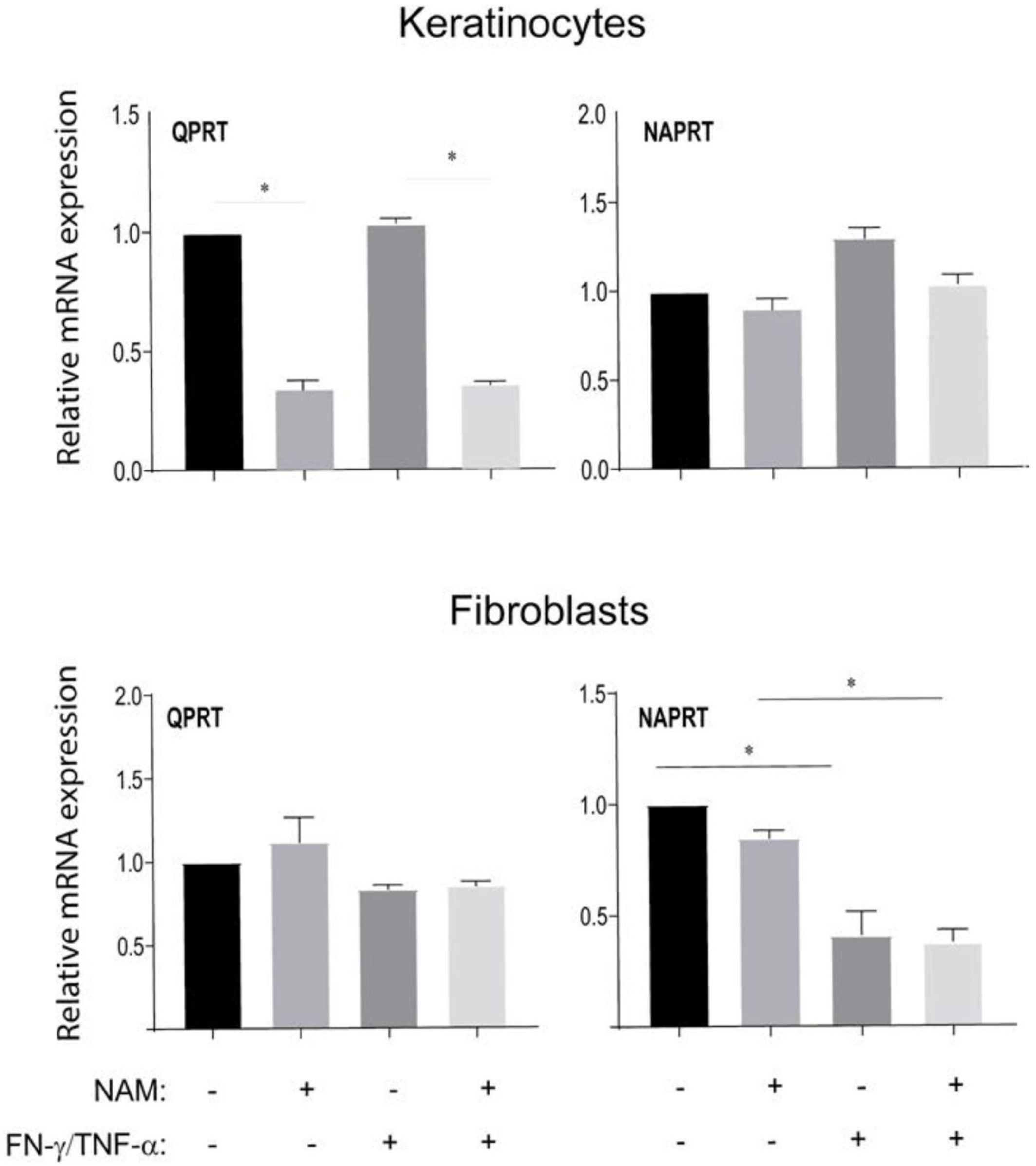

FIGURE S2

Figure S2. QPRT and NAPRT mRNA expression in keratinocytes and fibroblasts stimulated with NAM (1.5 mM) and IFN-γ/TNF-α for 18 h was detected by real-time PCR and normalized to HPRT1 levels. All data shown are the mean of three different experiments ± SD. *$p \leq 0.05$, calculated by paired Student's *t* test.

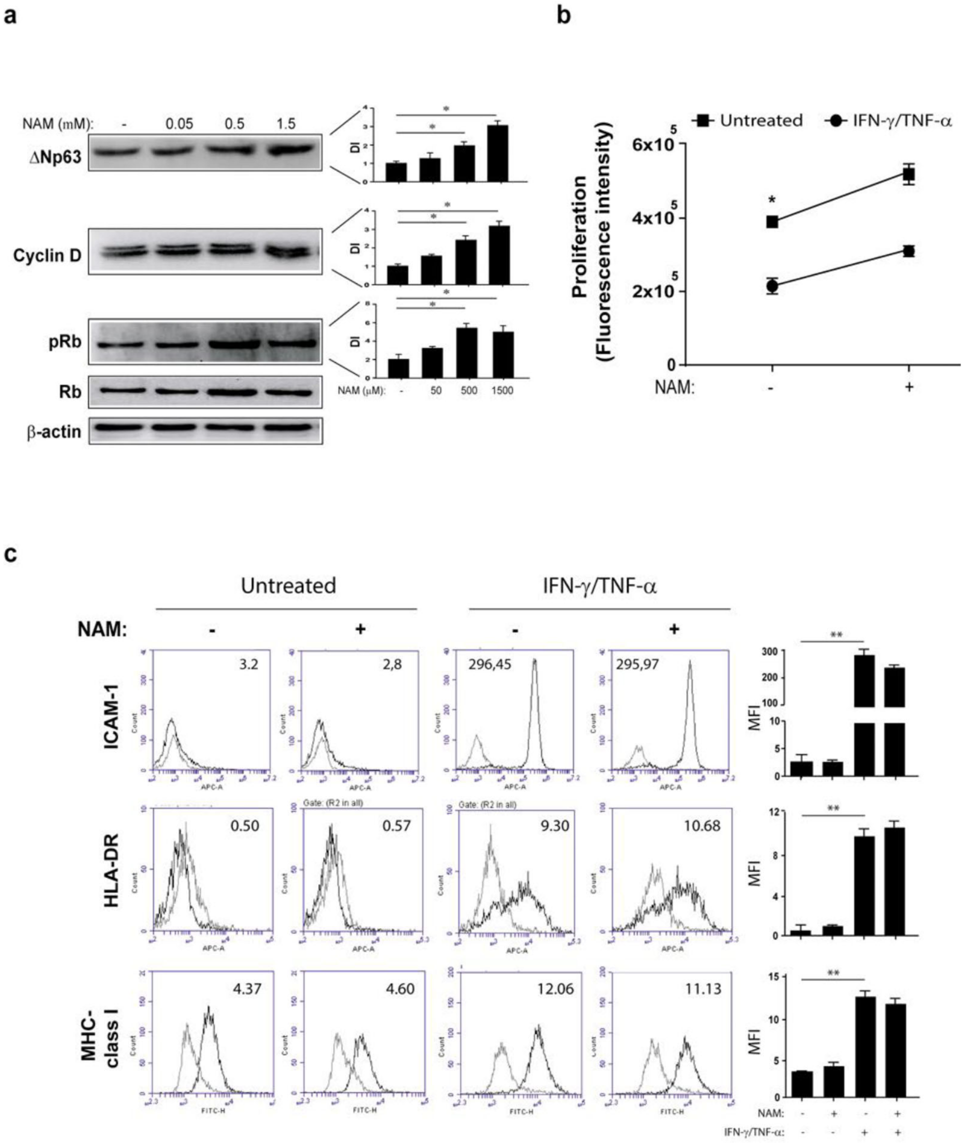

FIGURE S3

Figure S3. a Protein extracts were obtained from proliferating keratinocyte cultures exposed to increasing NAM doses for 48 h and subjected to WB analysis to detect ΔNp63, Cyclin D and pRb. β-actin was used as loading control. DI indicates the mean of densitometric intensity of three different WB ± SD of the indicated proteins normalized for β-actin shown in one representative WB. *$p \leq 0.05$ by paired Student's *t* test. **b** Cell proliferation was evaluated by CyQUANT assay on primary cultures of human keratinocytes in KBM left untreated or treated with increasing doses of NAM (1.5 mM) in presence or not of IFN-γ/TNF-α for 48 h. Data are expressed as mean of three independent experiments ± SD. *$p \leq 0.05$ was calculated by paired Student's *t* test. **c** ICAM-1, HLA-DR and MHC-class I expression was evaluated by flow cytometry analysis on keratinocytes stimulated for 24 h by IFN-γ/TNF-α and treated with NAM (1.5 mM). Data are shown as means of fluorescence intensity

(MFI). All data shown are the mean of three different experiments ± SD. **$p \leq 0.01$, calculated by paired Student's t test.

a

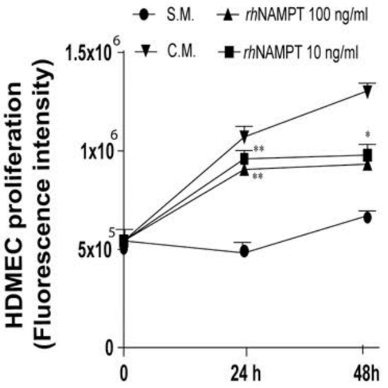

b

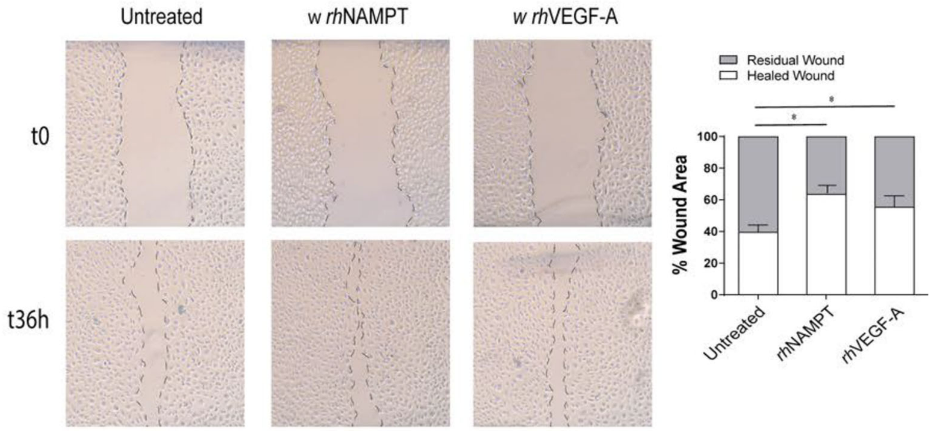

FIGURE S4

Figure S4. a CyQUANT proliferation assay was performed to determine proliferation of HDMEC grown in EGM complete medium (C.M.) or in EBM starvation medium (S.M.) in presence of rh NAMPT (10 ng/ml or 100 ng/ml) for 24 and 48 hours. Data are shown as the mean of fluorescence intensity values obtained from three independent experiments ± SD. *$p \leq 0.05$ and **$p \leq 0.01$, paired Student's t test. **b** Scratch assays were carried out on HDMEC grown in starvation medium, treated

with NAMPT (100 ng/ml), VEGF-A (50 ng/ml) for 36 h or left untreated. Microscopic images were taken immediately after and 36 h after wound induction on confluent cell layers. Initial scratches (0 h) are dashed grey. Cell-free area was measured and indicated as residual wound. Data are reported as healed wound (blank area of bars) *vs* residual wound (grey area of bars) which a value of 100% was given. Data are shown as mean of percentage values obtained from three independent experiments ± SD. *$p \leq 0.05$ was calculated by paired Student's t test.
